# Fixing the food environment: beyond weight-loss drugs

**DOI:** 10.1017/S1368980026101839

**Published:** 2026-02-06

**Authors:** Elisa Pineda, Charlotte Fenner, Cédric N. H. Middel

**Affiliations:** 1 The George Institute for Global Health UK, Imperial College London, UK; 2 School of Public Health, Faculty of Medicine, Imperial College London, UK; 3 Amsterdam UMC, location VUmc, Amsterdam, The Netherlands

**Keywords:** Food environment, Obesity, Ultra-processed foods, Health equity, Pharmacological treatments

## Abstract

The growing use of glucagon-like peptide-1 (GLP-1) receptor agonists and dual GIP/GLP-1 agonists has intensified debate over the role of pharmacotherapy in addressing obesity. While these drugs can support short-term weight loss, access remains limited, costly and unequal across health systems. Weight regain after cessation and recent price increases, such as for tirzepatide in the United Kingdom, underscore the fragility and inequity of drug-focused approaches. Reliance on medication risks diverts attention from structural drivers of obesity, including the widespread availability, marketing and placement of ultra-processed and high-fat, salt or sugar products and limited access to healthy, minimally processed foods. Population-level action, including mandatory reformulation, marketing restrictions, improved affordability and expanded access to nutritious foods, is essential. Medications may support individuals, but only comprehensive food-system reform can sustainably reduce obesity and diet-related disease.

The global rise in obesity and diet-related non-communicable diseases has sparked growing interest in pharmacological treatments. This is especially true of glucagon-like peptide-1 (GLP-1) receptor agonists such as semaglutide and dual glucose-dependent insulinotropic polypeptide (GIP)/GLP-1 agonists, such as tirzepatide, both now considered notable weight management solutions.^1^ But while they may offer short-term benefits for some, they come with limitations and should not be treated as substitutes for action on unhealthy food environments driving the obesity epidemic.^2,3^

## Access remains limited and unequal

GLP-1 and dual GLP-1/GIP receptor agonists work by enhancing insulin secretion, delaying gastric emptying and promoting satiety, resulting in weight loss. In the UK, National Institute for Health and Care Excellence (NICE) recommends them for adults with a BMI ≥35 kg/m^2^ and at least one weight-related comorbidity, or for those with a BMI >30 who meet specific referral criteria (e.g. unsuccessful conventional treatment). However, National Health Service (NHS)-funded access is only available for 2 years within specialist services.^1^ Without broader improvements to the food environment, weight regain after stopping treatment is a likely risk, and long-lasting outcomes will therefore remain poor.

Even for those who qualify, access remains inconsistent. This is not just a challenge unique to the UK. In the USA, high cost and strict insurance criteria make these treatments unaffordable for many, particularly lower-income groups who are already disproportionately affected by obesity, due to systemic issues such as food deserts and aggressive marketing. In Latin America, access is mostly restricted to the private sector and often unaffordable. This has fuelled a black market for counterfeit products, reported in Brazil, Mexico and Argentina, posing serious health risks. These examples highlight major limitations of these medications as a long-term solution, especially when factoring in known side effects and the lack of long-term data. Sustained use is costly, and broader action is still required.

Recent developments underline these access challenges. The announced 170% UK price increase for tirzepatide (*Mounjaro*) following US pressure to ‘equalise’ drug costs internationally illustrates the fragility and inequity of relying on pharmacological solutions.^4^ Although NHS-negotiated discounts may shield some patients, private patients, who currently make up most UK users, are likely to face steep cost rises. This shift not only risks widening inequalities in access, but also highlights how global political pressures can directly affect national affordability of obesity treatments. Such volatility further demonstrates why population-level food system interventions remain indispensable.

The NHS’s new 10-Year Plan expands access to weight-loss drugs through outcome-based models, but without bold public health reforms, such as mandatory reformulation, marketing restrictions and better access to healthy food, these efforts risk being palliative, not preventive.

## Rethinking and tackling the root causes

A healthy weight cannot be maintained without a healthier food environment and access to high-quality, timely information that enables informed choices. Clear, consistent front-of-pack food labelling, alongside effective lifestyle advice delivered in primary care, is essential but remain inconsistently implemented in the UK.^5,6^

Regulation of marketing, product placement and food composition is essential. This is especially true for products high in fat, salt or sugar and ultra-processed foods, which are heavily promoted to children.^7^ Evidence shows that limiting exposure to these foods can reduce unhealthy food purchases and support healthier diets across the population.^2,3^

Reformulating processed foods and shifting the balance of food availability towards healthier options in retail environments are key strategies.^8,9^ While some progress has been made, most notably the UK’s earlier success in reducing population salt intake, progress has now slowed and remains uneven, largely because reformulation efforts are voluntary and limited in scope. Governments must go further; they should mandate or incentivise reductions in salt, sugars, trans-fats and other harmful additives, alongside measures to reduce the dominance of unhealthy products on supermarket shelves. UPF continue to contain ingredients linked to inflammation, gut microbiome disruption and increased risk of chronic disease.^10–12^

Marketing and placement of unhealthy foods in stores, schools and at points of sale drive impulse purchases, especially of ultra-processed products.^13^ While the UK has begun to restrict such promotions and availability of unhealthy foods in schools has been restricted in the UK, gaps remain, particularly in protecting children. Recent evaluations highlight both the promise and the shortcomings of current policies, reinforcing the need for system-wide regulation.^14^

Improving access to sustainable, healthier, minimally processed foods must also be made a priority. Policies that improve the affordability and availability of fresh fruit, vegetables, whole grains, healthy fats and lean proteins, such as pulses, are fundamental. This includes shifting agricultural subsidies away from crops that fuel UPF production such as corn and soya and towards those that support healthier, more nutritious diets. In the UK, this is particularly important given that around 85% of land used to produce UK food is currently devoted to livestock grazing or to growing crops for animal feed rather than direct human consumption, limiting the supply and affordability of foods aligned with healthy dietary patterns.^15^

The NHS’s ‘Fit for the Future’ 10-Year Health Plan outlines a ‘moonshot to end the obesity epidemic’, including welcome steps such as banning energy drinks for children, restricting junk food ads and mandating health food sales reporting.^16^ However, it still lacks robust regulation of marketing practices across the wider food environment, including hospitals, the out-of-home sector (such as fast-food outlets and chain restaurants), delivery platforms and pervasive advertising and placement of ultra-processed products in everyday settings.

Without systemic changes to improve access to healthy foods, lower-income households will continue to rely on cheap, energy-dense options, locking them into cycles of poor nutrition and chronic disease. Medications like GLP-1s can help some individuals, but they cannot fix a broken system.

## Conclusions

Obesity cannot be addressed through individual treatment alone. A sustainable response requires population-level policies: tighter food marketing regulation, mandatory reformulation, clearer labelling and better access to whole foods.

Relying on these medications, with an expanding focus on oral and longer-acting formulations, further risks reducing obesity to a clinical issue, ignoring systemic drivers, aggressive marketing, widespread ultra-processed foods and industry resistance to regulation.

The NHS 10-Year Plan makes important commitments on prevention, school meals and marketing restrictions.^16^ But without addressing the affordability and accessibility of healthy diets, even the best treatments will fall short, leaving the underlying system unchanged and pressure on the NHS unchecked.
